# Stratifying the risk of anal high-grade squamous intraepithelial lesion among immunosuppressed women with genital HPV

**DOI:** 10.3389/fimmu.2026.1936298

**Published:** 2026-08-31

**Authors:** Isabel Matas, Maria Pifarré, Aureli Torné, Adela Saco, Katarzyna Darecka, Esther Guerra, Cristina Martí, Judit Bruch, Ada Cardiel, Natalia Rakislova, Lorena Marimon, Isabet Mayordomo-Bofill, Ingrid Victoria, Silvia Alós, Pere Fusté, Jaume Ordi, Marta del Pino

**Affiliations:** 1 Institute Clinic of Gynecology, Obstetrics, and Neonatology, Hospital Clínic, University of Barcelona, Barcelona, Spain; 2 Institut d’Investigacions Biomèdiques August Pi i Sunyer (IDIBAPS), Barcelona, Spain; 3 Department of Pathology, Hospital Clínic, University of Barcelona, Barcelona, Spain; 4 ISGlobal, Hospital Clínic, University of Barcelona, Barcelona, Spain

**Keywords:** anal intraepithelial neoplasia, high grade squamous intraepithelial lesion (HSIL), human immunodeficiency virus, human papillomavirus, immunosuppressed women

## Abstract

**Introduction:**

Immunosuppressed women with human papillomavirus (HPV)-relate7d genital disease are at increased risk of anal squamous cell carcinoma (ASCC), yet optimal risk stratification and screening strategies remain poorly defined. The aim of this study was to evaluate the prevalence of anal high-grade squamous intraepithelial lesions/anal intraepithelial neoplasia (HSIL/AIN), determine whether the anatomical distribution of genital HPV-associated disease provides clinically relevant information for risk stratification, and to assess the diagnostic performance of different screening strategies in this high-risk population.

**Methods:**

A total of 100 immunosuppressed women undergoing ASCC screening were included. All women underwent anal high-risk HPV testing and genotyping and liquid-based cytology, followed by high-resolution anoscopy when indicated.

**Results:**

Anal HPV infection was detected in 77.0% of women, and histologically confirmed HSIL/AIN was detected in 33.0% of the overall cohort. The proportion of women with HSIL/AIN differed according to the anatomical distribution of HPV-related genital lesions. Women with vulvar HSIL, either as isolated disease (52.6%) or as part of multizone HSIL (64.3%), showed the highest proportion of detected HSIL/AIN, whereas HSIL/AIN was detected less frequently among women with HPV infection without genital HSIL (22.2%) (p=0.037 and p=0.007, respectively). Anal HPV testing alone showed high sensitivity (97.0%; 95% CI: 84.2-99.9) and negative predictive value (95.7%; 95% CI: 78.1-99.9), whereas combined strategies slightly increased sensitivity at the expense of lower specificity and higher referral rates.

**Discussion:**

In conclusion, the anatomical distribution of HPV-associated genital disease appears to provide clinically relevant risk stratification. Women with vulvar HSIL represent a priority subgroup for ASCC screening. Anal HPV testing alone showed high sensitivity and NPV. These findings suggest that anal HPV testing may represent a useful primary screening approach in this population.

## Introduction

Anal squamous cell carcinoma (ASCC) is an increasingly important public health problem, with incidence rates rising steadily over recent decades (1). The pathogenesis of ASCC closely parallels that of cervical cancer. Both malignancies are predominantly driven by high-risk human papillomavirus (HPV) and are preceded by high-grade squamous intraepithelial lesions (HSIL), which share similar morphological features at both anatomical sites (2). This biological relationship has been extensively reviewed in the context of cervical carcinogenesis and HPV-associated precancerous lesions (3).

Although it has traditionally been considered a disease affecting mainly men who have sex with men and people living with human immunodeficiency virus (HIV), more recent epidemiological evidence has shown that additional population groups, particularly women receiving immunosuppressive therapy, including solid organ or hematopoietic stem cell transplant recipients, as well as individuals with inflammatory bowel disease or other autoimmune conditions also have a substantially increased risk of ASCC (4, 5). In addition, women with HPV-associated premalignant or malignant lesions of the lower genital tract are at increased risk of ASCC, even in the absence of systemic immunosuppression (4, 6, 7).

The publication of the anal cancer/HSIL outcomes research (ANCHOR) trial in 2022 represented a major advance in the field by showing that treatment of HSIL/anal intraepithelial lesion (AIN) significantly reduced progression to ASCC among people living with HIV (8–10). More recently, in 2024, the International Anal Neoplasia Society (IANS) issued consensus guidelines recommending ASCC screening in several high-risk populations, including women living with HIV (WLWH) aged 45 years or older and solid organ transplant recipients beginning 10 years after transplantation; the guidelines also support shared decision-making in selected intermediate-risk groups, such as individuals with other forms of immunosuppression (11). Previous studies have also shown that women with HPV-associated disease of the lower genital tract are at increased risk of anal HPV infection, HSIL/AIN, and ASCC (7). However, most available studies have evaluated these risk factors independently, and limited evidence is available on women in whom immunosuppression and genital HPV-associated disease coexist (12).

These landmark studies have established the current framework for anal cancer screening in high-risk populations but have also highlighted the limited evidence supporting the optimal screening strategy in specific subgroups, particularly immunosuppressed women with HPV-associated genital disease. Current guidelines recommend anal HPV testing, anal cytology, or cotesting (HPV testing and anal cytology) for ASCC screening. However, the optimal strategy for using these tests across different high-risk groups remains insufficiently validated (9–11, 13). A major challenge in ASCC prevention is balancing the very high sensitivity of HPV testing with its limited specificity, especially in some high-risk populations, which may generate unsustainable referral rates for high-resolution anoscopy (HRA). Thus, in classically high-risk populations such as WLWH and immunosuppressed women, the exceptionally high prevalence of anal HPV infection leads to excessive referral rates for HRA if HPV testing is used as the primary screening strategy. In contrast, a recent study conducted in immunocompetent women by our group has shown that HPV testing alone is the most effective strategy in this population (14). Importantly, the existing recommendations classify high-risk groups as discrete risk categories according to the presence of individual conditions such as HIV infection, transplantation, or genital HPV-associated disease. In clinical practice, however, these risk factors often overlap. Furthermore, little is known about whether the anatomical distribution of HPV-associated genital disease, i.e., cervical, vaginal, vulvar, or multizone involvement, provides additional information for ASCC risk stratification. Better risk stratification could optimize HRA referral and refine screening algorithms, thereby improving resource allocation.

The aim of the present study was to evaluate the prevalence of anal HSIL/AIN among immunosuppressed women with HPV-associated genital disease, to determine whether the anatomical distribution of genital HSIL provides relevant information on the risk of anal disease and could help in better stratifying immunosuppressed patients. We also aimed to assess the diagnostic performance of the currently available screening tests in this high-risk population.

## Materials and methods

### Selection criteria

We conducted a single-center cross-sectional study at the lower anogenital tract disease unit of the Hospital Clínic of Barcelona, a tertiary hospital that serves as a referral center for women with HPV-associated disease of the lower genital tract. Since 2019, ASCC screening has been offered to immunosuppressed women aged ≥30 years with genital HPV infection and/or HPV-associated lesions of the lower genital tract.

Our study included all consecutive women referred to our unit between March 2019 and September 2024 who fulfilled the following criteria: a) age ≥30 years; b) immunosuppression of any cause; and c) HPV infection and/or HPV-associated lesions involving the lower genital tract. Exclusion criteria included: a) pregnancy; b) prior ASCC; and c) treatment of HSIL/AIN within the previous three years.

All women included in the study were receiving treatment for their underlying condition. WLWH were receiving antiretroviral therapy, whereas transplant recipients and women with other immunosuppressive conditions were receiving immunosuppressive or disease-modifying treatment. Detailed information on the specific drugs, treatment duration, and CD4 cell counts was not systematically recorded.

### Lower genital tract evaluation and genital HSIL treatment

At the referral visit, all women underwent a systematic evaluation of the entire lower anogenital tract, including thorough assessment of the cervix, vagina, vulva, perineum, and perianal region. Cervical samples were obtained for HPV testing and liquid-based cytology (LBC). Colposcopy was performed according to institutional protocols in women with: a) a positive result for HPV16/18; b) persistent infection by high-risk HPV other than 16/18 (i.e., two positive HPV test results separated by at least one year); c) cervical LBC showing LSIL or HSIL.

Women fulfilling these criteria underwent systematic colposcopic examination of the cervix and the vagina with directed biopsies of any suspected lesion. Additionally, visible lesions of the vulva, perineum, or perineal area, were biopsied using a 3- or 4-mm punch biopsy for histological assessment.

The management of the genital lesions followed standard institutional protocols: women with histologically confirmed cervical HSIL underwent excisional treatment under colposcopic guidance (15). Vaginal HSIL was treated with thermocoagulation or laser vaporization under colposcopic visualization. Women with vulvar or perineal/perianal HSIL underwent either excisional treatment, topical imiquimod, or ablative therapies after excluding invasive disease (16).

### ASCC screening, high-resolution anoscopy, and anal HSIL diagnosis and management

Anal samples were collected using a cytobrush. The brush was inserted 3–4 cm into the anal canal and rotated 360° clockwise for 30 seconds, followed by a 360° counterclockwise rotation for an additional 30 seconds. Samples were preserved in PreservCyt (Hologic, Marlborough, MA, USA), and used for HPV testing and LBC, which were performed in all cases.

Referral to HRA was indicated in any women with a positive anal HPV test (any genotype) and/or an abnormal result of the anal LBC. Women with an unsatisfactory anal LBC result and a negative HPV test underwent repeated cytology. Persistently unsatisfactory cytology prompted HRA referral.

HRA was conducted by an experienced gynecologist trained in the technique. Prior to the procedure, a digital anorectal examination was systematically performed to exclude abnormalities suspicious for ASCC (11). HRA was conducted using an Olympus Evis Exera II CV-180 anoscope (Olympus, Barcelona, Spain). A 5% acetic acid solution was applied for 2 minutes followed by iodine solution to enhance abnormal areas. Directed biopsies were obtained from all suspicious areas. In the absence of visible lesions, HRA was considered negative for disease.

Women with both negative anal high-risk HPV test and negative anal LBC did not meet referral criteria for HRA, and therefore, did not undergo HRA for histological verification. For the purposes of the screening strategy analysis, these women were considered negative for HSIL/AIN. Women diagnosed with histological HSIL/AIN were managed according to institutional protocols (6).

### HPV testing and liquid‐based cytology

Cervical and anal specimens were analyzed using the Cobas^®^ HPV assay (Roche Molecular Diagnostics, Mannheim, Germany), which detects 14 high-risk HPV types, and provides individual genotyping for HPV 16 and HPV 18, whereas the remaining genotypes are reported as “other high-risk HPV”.

LBC slides were prepared using the ThinPrep T2000 slide processor (Hologic) and interpreted according to the Bethesda system (17).

### Histological processing and diagnosis

All biopsies and surgical specimens were formalin-fixed, paraffin-embedded, sectioned at 3 μm, and stained with hematoxylin and eosin (H&E). p16 immunohistochemistry (IHC) was performed in all cases using the Roche platform and the CINtec Histology Kit (clone E6H4; Roche-Mtm Laboratories, Heidelberg, Germany). Only cases showing continuous “block” staining of the basal and parabasal layers were considered positive for p16 (18, 19). In all vulvar, perineal/perianal and anal biopsies p53 IHC staining (clone DO-7; Dako, Carpinteria, CA) was also performed. p53 IHC was interpreted according to pattern-based criteria (20).

Histological diagnosis was primarily based on morphology. p16 immunohistochemistry was mainly applied to distinguish HSIL from its mimickers (atrophy, reparative changes), as recommended by the WHO (18). The diagnosis of HSIL was based primarily on morphological criteria, and supported by block-positive p16 staining when indicated. The diagnosis of LSIL was also based on morphological criteria. p16 staining was only used as an adjunct in the diagnosis of LSIL vs. HSIL in morphologically equivocal cases, but did not result in systematic upgrading of LSIL lesions. p53 IHC was used to exclude HPV-independent lesions mimicking HSIL. In contrast to HPV-associated HSIL, these lesions typically show p16 negativity together with a mutation-type p53 staining pattern (21). Histological samples were classified as negative, LSIL or HSIL (17). The anatomical area and the grade of the lesion were indicated following the WHO terminology (22). Missing anal histological results were categorized as not available.

### Patient categorization

Women were categorized according to the anatomical distribution of HPV-associated genital HSIL in four predefined groups: 1) HPV infection, which included women with genital warts, and/or genital HPV infection without histologically confirmed genital HSIL. 2) cervical HSIL, which included women with isolated cervical or vaginal HSIL, which were analyzed together because they arise in closely histologically similar epithelia (non-keratinizing squamous epithelium) and are expected to have similar biological behavior; in addition, the small number of women with isolated vaginal HSIL precluded a separate analysis. 3) vulvar HSIL, comprising women with isolated vulvar or perineal HSIL. 4) multizone HSIL, which included women with HSIL involving two or more lower genital tract sites (cervix, vagina, vulva, and/or perineum/perianal region).

Immunosuppressed women were categorized into three groups: 1) WLHIV, 2) transplant recipients, including solid organ transplant recipients and hematopoietic stem cell transplant recipients, and 3) women with other autoimmune conditions.

In terms of HPV infection, samples were classified into three mutually exclusive, hierarchical categories: 1) HPV 16 (alone or with any other type, including HPV18); 2) HPV18 (alone or with HPV types other than 16/18); 3) HPV types other than 16/18. For the HPV types other than 16/18, type-specific information was not available and single infections could not be distinguished from multiple infections involving HPV types other than 16/18.

For analytical purposes, all patients from the group HPV/non-HSIL were grouped into a single category in the uterine cervix, independently of the result of the LBC (negative, ASC-US or LSIL). In contrast, in the anus, patients with a cytological result of ASC-US and those with LSIL, were analyzed as two distinct groups because of their relative high prevalence among women with negative anal HPV testing. Cytological results of atypical squamous cells, cannot exclude HSIL (ASC-H) were grouped with HSIL both in the cervix and the anal canal given their significant risk of underlying histological HSIL (23).

### Data analysis

The STATA/BE 18.0 software (College Station, Texas 77845 USA) was used for data analysis. Epidemiological and clinical variables including age, smoking, and HPV vaccination status prior to the referral visit were recorded.

Categorical variables were reported as absolute numbers and percentages and compared using the Chi-squared test or Fisher’s exact test, as appropriate. Continuous variables were reported as means and standard deviations (SD) and compared using analysis of variance (ANOVA). For HPV genotype concordance analyses, cervical and anal HPV results are compared at the genotype-category level using the hierarchical classification (HPV16, HPV18 and high-risk HPV types other than 16/18). Cervical/anal concordance was defined as the presence of at least one common HPV genotype in both anatomical sites (genital and anal sites). Analyses focused on HPV16 and HPV18; infections classified as HPV16 were considered concordant if HPV16 was detected at both sites, irrespective of additional co-infecting types.

Univariable logistic regression models were used to estimate the risk of histologically confirmed HSIL/AIN according to age, smoking status, study group, cervical and anal HPV type and anal LBC result. Risk estimates were expressed as odds ratios (ORs) and 95% confidence intervals (CIs). Variables associated with HSIL/AIN in univariable analyses (p<0.10) were entered into multivariable logistic regression models. The model included the main clinical characteristics evaluated in the study: anatomical distribution of genital disease, anal HPV result, and anal cytology. Multivariable logistic regression models were fitted to evaluate the independent association between the aforementioned variables and HSIL/AIN, while adjusting for potential confounders. The genital HPV type served as the reference category in all regression analyses. A p value of 0.05 was considered statistically significant.

For screening strategy analyses, we calculated the sensitivity, specificity, positive predictive value (PPV) and negative predictive value (NPV) for the detection of HSIL/AIN, with their corresponding 95% confidence intervals (95% CI), together with the proportion of women referred to HRA under each screening strategy. Women who met the referral criteria for HRA but declined the procedure were included in the denominator used to calculate the proportion of women meeting the criteria for referral to HRA, but were classified as having no available histological outcome and were excluded from all the analyses requiring histological confirmation.

## Results

### Study population and baseline characteristics

A total of 100 immunosuppressed women were included: 38 WLHIV, 25 transplant recipients (24 solid organ transplant recipients and 1 hematopoietic stem cell transplant recipient), and 37 women with other immunosuppressive conditions. The latter group included women with systemic lupus erythematosus (n=12), inflammatory bowel disease (n=7), systemic autoimmune or rheumatologic diseases (n=10), primary immunodeficiency syndromes (n=3), autoimmune hepatobiliary disease (n=2), hematologic malignancy (n=2), and multiple sclerosis (n=1).

According to the anatomical distribution of genital HPV-related disease, 45 women comprised the HPV infection group. The cervical HSIL group included 22 patients (17 with cervical lesions and 5 with vaginal lesions), the vulvar HSIL group included 19 women, and the multizone HSIL group 14 patients (13 of them with vulvar involvement). Baseline characteristics are shown in **Table 1**. Cervical HPV genotype distribution differed across the groups, with HPV16 more frequently detected among women with multizone HSIL and those with cervical HSIL (p=0.001). Abnormal cervical LBC results also differed across groups, with the highest proportion of HSIL cytology results observed in the group of patients with multizone HSIL, followed by those with cervical HSIL (p<0.001).

**Table 1:** Immunosuppression status, epidemiological characteristics and pathological results (HPV testing and liquid-based cytology [LBC]) in the cervical sample results across the five HPV-related lesion study groups included in the study.

Variable	HPV infection (n=45)		Cervical HSIL (n=22)		Vulvar HSIL (n=19)		Multizone HSIL (n=14)		
	n	(%)	n	(%)	n	(%)	n	(%)	p-value
Mean age (SD)	47.7	(10.7)	47.8	(10.4)	48.1	(10.3)	46.4	(8.3)	0.749
Immunosuppression status									
Women living with HIV	21	(46.7)	6	(27.3)	7	(36.8)	4	(28.6)	0.148
Transplant recipients	14	(31.1)	2	(9.1)	4	(21.1)	5	(35.7)	
Other conditions*	10	(22.2)	14	(63.7)	8	(42.1)	5	(35.7)	
Smoking habit									0.678
Non-smoker	17	(37.8)	9	(40.9)	6	(31.6)	4	(28.6)	
Ex-smoker	13	(28.9)	7	(31.8)	5	(26.3)	2	(14.3)	
Smoker	15	(33.3)	6	(27.3)	8	(42.1)	8	(57.1)	
Cervical HPV									0.001
Negative	11	(24.4)	0	(0.0)	8	(42.1)	2	(14.3)	
HPV16	6	(13.3)	10	(45.5)	7	(36.9)	9	(64.3)	
HPV18	3	(6.7)	1	(4.5)	0	(0.0)	0	(0.0)	
HPV other than 16/18	25	(55.6)	11	(50.0)	4	(21.1)	3	(21.4)	
Cervical LBC									<0.001
Negative	14	(31.1)	2	(9.1)	11	(57.9)	2	(14.3)	
ASC-US/LSIL	30	(66.7)	10	(45.5)	8	(42.1)	3	(21.4)	
HSIL	1	(2.2)	10	(45.5)	0	(0.0)	9	(64.3)	

HPV, human papillomavirus; HSIL, high-grade squamous intraepithelial lesion; LSIL, low-grade squamous intraepithelial lesion; ASC-US, atypical squamous cells of undetermined significance; HIV, human immunodeficiency virus.

* Other conditions included; systemic lupus erythematosus (n=12), inflammatory bowel disease (IBD) (n=7), polymyalgia rheumatica (n=2), psoriasis (n=2), autoimmune hepatitis (n=2), Sjögren’s syndrome (n=2), Severe Combined Immunodeficiency (SCID) (n=1), hypogammaglobulinemia (n=1), primary CD4 deficiency (n=1), Multiple Myeloma (MM) (n=1), Monoclonal Gammopathy of Undetermined Significance (MGUS) (n=1), sarcoidosis (n=1), psoriatic arthritis (n=1), Multiple Sclerosis (MS) (n=1), Mixed connective tissue disease (MCTD) (n=1), autoimmune anemia (n=1).

Values are presented as absolute numbers and percentages.

Statistically significant differences are highlighted in bold.

### Anal HPV testing and cytological results

**Table 2** summarizes anal HPV testing and LBC results in the different study groups. Overall, 77 of 100 women had a positive anal HPV test result. Anal HPV genotype distribution did not differ significantly across study groups (p=0.063), although HPV16 tended to be more frequent among women with vulvar HSIL (10/19; 52.6%) and multizone HSIL (8/14; 57.1%) than among women with HPV infection (11/45; 24.4%) or those with cervical HSIL (6/22; 27.3%). Anal LBC was unsatisfactory in 9/100 women. Among the 91 women with valid anal LBC, 57 (62.6%) showed abnormal results (≥ASC-US) and no differences were observed across the study groups (p=0.262). Anal HPV testing results (negative, HPV16, HPV18, HPV other than 16/18) and anal LBC results were also similar across the different immunosuppression categories (p=0.167 and p=0.376, respectively).

**Table 2:** Anal human papillomavirus (HPV) prevalence, anal liquid-based cytology [LBC] and anal histological results across the five HPV-related lesion study groups included in the study.

Anal test result	HPV infection (n=45)		Cervical HSIL (n=22)		Vulvar HSIL (n=19)		Multizone HSIL (n=14)		
	n	(%)	n	(%)	n	(%)	n	(%)	p-value
Anal HPV testing result									0.063
Negative	10	(22.2)	3	(13.6)	6	(31.6)	4	(28.6)	
HPV 16	11	(24.4)	6	(27.3)	10	(52.6)	8	(57.1)	
HPV 18	5	(11.1)	2	(9.1)	1	(5.3)	0	(0.0)	
HPV other than 16/18	19	(42.2)	11	(50.0)	2	(10.5)	2	(14.3)	
Anal liquid-based cytology result									0.262
Negative	16	(35.6)	10	(45.5)	6	(31.6)	2	(14.3)	
ASC-US	7	(15.6)	3	(13.6)	2	(10.5)	2	(14.3)	
LSIL	13	(28.9)	5	(22.7)	8	(42.1)	6	(42.9)	
HSIL	2	(4.4)	3	(13.6)	2	(10.5)	4	(28.6)	
Unsatisfactory	7	(15.6)	1	(4.6)	1	(5.3)	0	(0.0)	
Anal biopsy result									<0.001
Negative*	25	(55.6)	11	(50.0)	8	(42.1)	4	(28.6)	
LSIL	10	(22.2)	2	(9.1)	0	(0.0)	1	(7.1)	
HSIL	10	(22.2)	4	(18.2)	10	(52.6)	9	(64.3)	
NA	0	(0.0)	5	(22.7)	1	(5.3)	0	(0.0)	

HPV, human papillomavirus; HSIL, high-grade squamous intraepithelial lesion; LSIL, low-grade squamous intraepithelial lesion; ASC-US, atypical squamous cells of undetermined significance; NA, not available, missing values correspond to women who had an indication for HRA but did not undergo the procedure.

* Both women who did not meet the HRA referral criteria (women with negative anal HPV testing and negative anal LBC) and women who underwent HRA with no visible lesion (and therefore no biopsy) were classified as negative anal histology. Values are presented as absolute numbers and percentages.

Statistically significant differences are highlighted in bold.

Among the 67 women with concurrent cervical and anal HPV infection, genotype-category concordance was 85.2% (23/27) for HPV16, 50.0% (1/2) for HPV18, and 68.4% (26/38) for HPV types other than 16/18.

### High-resolution anoscopy and histological outcomes

**Figure 1:**
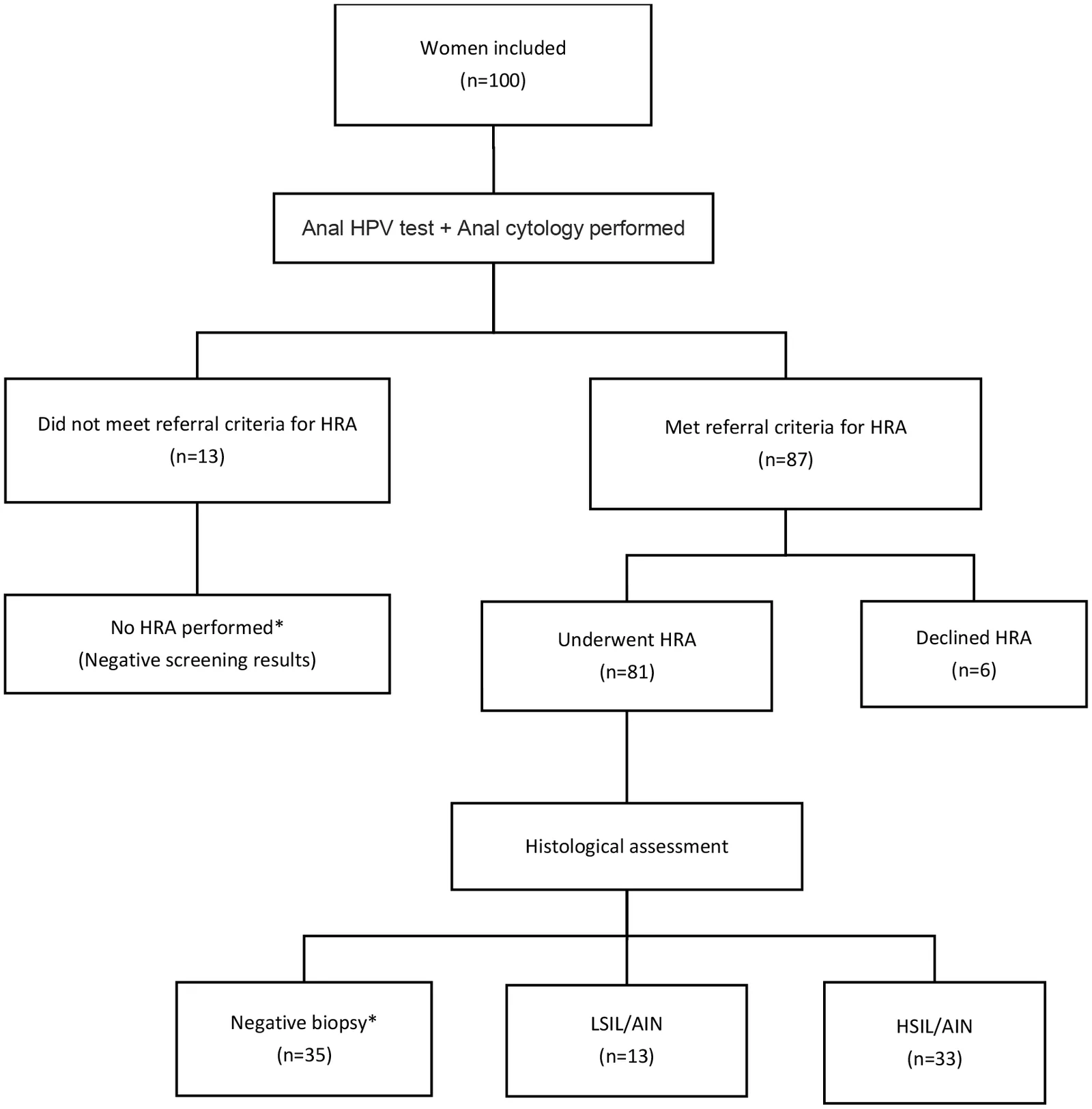
Study flow diagram of patient inclusion, referral for high-resolution anoscopy (HRA), HRA completion, and final diagnostic outcomes. ^*^Women who did not meet the referral criteria for HRA, as well as those who underwent HRA and had either a negative biopsy or no visible lesions on HRA, were classified as negative for HSIL/AIN (n=48).

Eighty-seven out of 100 women met the criteria for HRA referral. Of these, 81/87 (93.1%) underwent HRA, whereas six (6.9%) declined the procedure (two with anal HPV16, one with anal HPV18, and three with HPV other than 16/18). Histologically confirmed HSIL/AIN was diagnosed in 33/81 (40.7%) women who underwent HRA, corresponding to 33/100 (33.0%) women in the overall cohort. Because not all participants underwent HRA, the latter proportion represents detected HSIL/AIN rather than an estimate of the true prevalence. A flow diagram summarizing patient inclusion, HRA referral, HRA completion and final diagnosis is shown in **Figure 1**.

Mean age of women diagnosed with HSIL/AIN was 46.5 years (SD 9.7), ranging from 30.0 to 70.7 years. Although not statistically significant, women with HSIL/AIN in vulvar or multizone HSIL groups tended to be younger than those in cervical HSIL group (43.8 and 43.4, vs. 59.2 years, respectively; Kruskal–Wallis p=0.080). The prevalence of HSIL/AIN differed significantly according to the anatomical distribution of HPV-associated genital disease (**Table 2**). HSIL/AIN was more frequent among women with vulvar HSIL (10/19; 52.6%; p=0.037) and those with multizone HSIL (9/14; 64.3%; p=0.007) compared with the HPV infection group (10/45; 22.2%). When women with vulvar involvement, either as isolated disease (vulvar HSIL group) or as part of a multicentric disease (multizone HSIL group) were considered together (n=32), anal HSIL/AIN was detected in 19/32 (59.4%), a proportion significantly higher than the HPV infection group (p=0.001).

The histological diagnoses stratified according to anal HPV testing and LBC results are shown in **Table 3**. HSIL/AIN was detected in 32/71 (45.1%) women with a positive anal HPV test result who underwent HRA. Within this HPV-positive group, the proportion of HSIL/AIN increased with cytological severity from 20.0% (4/20) in women with negative cytology to 80.0% (8/10) among those with HSIL cytology (p<0.001). Among women with negative anal HPV testing who underwent HRA, HSIL/AIN was identified in 1/23 (4.3%), corresponding to a woman with LSIL cytology. Histological HSIL/AIN was diagnosed in 18/35 (51.4%) women with anal HPV16. Sixteen of these 18 women (88.9%) had concurrent infection with additional HPV types: 12 women had an HPV type other than HPV16/18 and 4 also had HPV18.

**Table 3:** Anal histological results stratified by anal human papillomavirus (HPV) test and anal liquid-based cytology (LBC) results.

		Anal histological results				
		Negative^*^	LSIL/AIN	HSIL/AIN	NA	
Anal HPV	Anal LBC	n (%)	n (%)	n (%)	n (%)	p-value
Negative	Negative	13 (100.0)	0 (0.0)	0 (0.0)	0 (0.0)	NA
	ASC-US	2 (100.0)	0 (0.0)	0 (0.0)	0 (0.0)	
	LSIL	2 (40.0)	2 (40.0)	1 (20.0)	0 (0.0)	
	HSIL	1 (100.0)	0 (0.0)	0 (0.0)	0 (0.0)	
	Unsatisfactory	2 (100.0)	0 (0.0)	0 (0.0)	0 (0.0)	
Positive	Negative	13 (65.0)	0 (0.0)	4 (20.0)	3 (15.0)	**<0.001**
	ASC-US	5 (41.7)	2 (16.7)	4 (33.3)	1 (8.3)	
	LSIL	5 (17.9)	9 (32.1)	14 (50.0)	0 (0.0)	
	HSIL	1 (10.0)	0 (0.0)	8 (80.0)	1 (10.0)	
	Unsatisfactory	4 (57.1)	0 (0.0)	2 (28.6)	1 (14.3)	

Values are presented as absolute numbers and percentages.

HPV, human papillomavirus; LSIL, low-grade squamous intraepithelial lesion; ASC-US; atypical squamous cells of undetermined significance; HSIL, high-grade squamous intraepithelial lesion; LSIL/AIN, anal low-grade squamous intraepithelial lesion; HSIL/AIN, anal high-grade squamous intraepithelial lesion; NA, not available, missing values correspond to women who had an indication for HRA but did not undergo the procedure.

* Both women who did not meet the HRA referral criteria (women with negative anal HPV testing and negative anal LBC) and women who underwent HRA with no visible lesion (and therefore no biopsy) were classified as negative anal histology.

Statistically significant differences are highlighted in bold.

### Factors independently associated with anal HSIL/AIN

**Table 4** summarizes the univariable and multivariable analyses of factors associated with histological HSIL/AIN. In the univariable analysis, vulvar HSIL, multizone HSIL, positive anal HPV infection (HPV16 and HPV other than 16/18 compared with negative anal HPV) and abnormal anal LBC result (LSIL and HSIL compared with negative cytology) were associated with increased risk of HSIL/AIN. In the multivariable model, vulvar HSIL (OR 7.0; 95% CI 1.3-37.2), multizone HSIL (OR 8.5; 95% CI 1.2-61.0) remained independently associated with HSIL/AIN. Positive anal HPV testing (HPV16 [OR 35.0; 95% CI 3.0-406.4] and HPV other than 16/18 [OR 42.8; 95% CI 3.1-591.5]) and anal HSIL cytology (OR 13.7; 95% CI 1.6-116.7) also remained independently associated with HSIL/AIN.

**Table 4:** Univariate and multivariate logistical regression analysis of risk factors associated with the diagnosis of high grade squamous intraepithelial lesion/anal intraepithelial neoplasia (HSIL/AIN) in immunosuppressed women.

Variable	Univariable analysis			Multivariable analysis		
	OR	(95 CI %)	p-value	OR	(95 CI %)	p-value
Age	0.9	(0.9-1.0)	0.424			
Smoking habits						
Non smoker	1					
Ex-smoker	1.0	(0.3-2.8)	0.937			
Smoker	1.4	(0.5-3.7)	0.513			
Study group						
HPV infection	1			1		
Cervical HSIL	2.4	(0.7-16.0)	0.361			
Vulvar HSIL	3.9	(1.2-12.2)	**0.020**	7.0	(1.3-37.2)	**0.023**
Multizone HSIL	6.3	(1.7-23.1)	**0.006**	8.5	(1.2-61.0)	**0.033**
Cervical HPV						
Negative	1			1		
HPV 16	1.3	(0.4-4.3)	0.658			
HPV 18	1	*				
HPV other than 16/18	1.5	(0.5-4.6)	0.496			
Anal HPV						
Negative	1			1		
HPV 16	23.3	(2.8-192.3)	**0.003**	35.0	(3.0-406.4)	**0.005**
HPV 18	3.1	(0.2-57.1)	0.439			
HPV other than 16/18	13.6	(1.6-113.5)	**0.016**	42.8	(3.1-591.5)	**0.005**
Anal LBC						
Negative	1			1		
ASC-US	3.0	(0.6-14.3)	0.167			
LSIL	6.6	(1.9-23.2)	**0.003**			
HSIL	20.0	(3.7-108.2)	**0.001**	13.7	(1.6-116.7)	**0.017**
Unsatisfactory	2.1	(0.3-14.1)	0.428			

OR, odds ratio; CI, confidence interval. HPV, human papillomavirus; LSIL, low-grade squamous intraepithelial lesion; ASC-US; atypical squamous cells of undetermined significance; HSIL, high-grade squamous intraepithelial lesion.

*Estimates for cervical HPV18 could not be calculated due to sparse data.

Statistically significant differences are highlighted in bold.

### Clinical performance of different screening strategies

**Table 5** summarizes the clinical performance of the different screening strategies for HSIL/AIN detection. The screening strategy based on positive hrHPV testing or abnormal cytology achieved 100% sensitivity (95% CI: 89.4-100) and negative predictive value (95% CI: 79.4-99.9), although at the expense of the lowest specificity (23.9%; 95% CI: 14.3-35.9) and the highest referral rate to HRA (87.0%). Notably, HPV testing alone showed a comparable sensitivity (97.0%; 95% CI: 84.2-99.9) while improving specificity (32.8%; 95% CI: 21.8-45.4) and reducing referrals to HRA (77.0%). Cytology alone (≥ASC-US) substantially reduced referrals to HRA (57.0%) but at the cost of a lower sensitivity (87.1%; 95% CI: 70.2-96.4). Interestingly, among anal HPV-positive women, anal cytology provided additional risk stratification, with the proportion of women with HSIL/AIN increasing from 20.0% in women with negative cytology to 80.0% in those with HSIL cytology.

**Table 5:** Sensitivity and specificity of the anal HPV, anal cytology, and anal co-test (HPV and cytology) for the diagnosis of HSIL/AIN in immunosuppressed women.

Screening strategy	Sensitivity	(95% CI)	Specificity	(95% CI)	PPV	(95% CI)	NPV	(95% CI)	Referral to HRA, n (%)	HSIL/AIN missed n (%)*
Positive HPV OR cytology ≥ASC-US (*study strategy*)	100%	(89.4-100)	23.9%	(14.3-35.9)	39.3%	(28.8-50.5)	100%	(79.4-99.9)	87 (87.0)	0 (0.0)
Positive HPV testing alone (any type)	97%	(84.2-99.9)	32.8%	(21.8-45.4)	41.6%	(30.4-53.4)	95.7%	(78.1-99.9)	77 (77.0)	1 (3.0)
Cytology result ≥ASC-US alone	87.1%	(70.2-96.4)	50.0%	(36.8-63.2)	47.4%	(34.0-61.0)	88.2%	(72.5-96.7)	57 (57.0)	4 (12.1)
HPV16/types other than 16/18 AND cytology ≥ASC-US	84.8%	(68.1-94.9)	56.7%	(44.0-68.8)	49.1%	(35.6-62.7)	88.4%	(74.9-96.1)	57 (57.0)	5 (15.2)
Positive HPV AND cytology ≥ASC-US	78.8%	(61.1-91.0)	64.2%	(51.5-75.5)	52.0%	(37.4-66.3)	86.0%	(73.3-94.2)	50 (50.0)	7 (21.2)
Positive HPV16 OR HPV18	57.6%	(39.2-74.5)	64.2%	(51.5-75.5)	44.2%	(29.1-60.1)	75.4%	(62.2-85.9)	43 (43.0)	14 (42.4)
Positive HPV16 OR 18 AND cytology ≥ASC-US	48.5%	(30.8-66.5)	82.1%	(70.8-90.4)	57.1%	(37.2-75.5)	76.4%	(64.9-85.6)	28 (28.0)	17 (51.5)

PPV, positive predictive value; NPV, negative predictive value; CI, confidence interval; HPV, human papillomavirus; LBC, liquid-based cytology. Abnormal LBC includes, LSIL, ASC-US and HSIL LBC results. LSIL, low-grade squamous intraepithelial lesion; ASC-US; atypical squamous cells of undetermined significance; HSIL, high-grade squamous intraepithelial lesion. HRA, high resolution anoscopy. * % of 33 HSIL/AIN diagnosed in the study cohort.

## Discussion

The main finding of this study is that, among immunosuppressed women, the anatomical distribution of the HPV-associated genital disease emerged as the clinical factor most strongly associated with an increased risk of anal HSIL/AIN. Women with vulvar HSIL, either as isolated disease or as part of multizone disease, showed a markedly higher prevalence of anal HSIL/AIN compared with women with isolated cervical HSIL or HPV infection without HSIL in the lower genital tract.

In this study focused on immunosuppressed women with HPV-associated lower genital tract disease, we observed a high burden of anal HPV infection (77.0%), and histologically confirmed HSIL/AIN was detected in 33.0% of the overall cohort. Remarkably, anal HSIL/AIN prevalence varied markedly according to the anatomical distribution of genital HPV-associated disease, with rates above 50% in women with multizone HSIL (64.3%) and vulvar HSIL (52.6%). In contrast, in our cohort, no differences in HSIL/AIN prevalence were observed across immunosuppression categories. These findings may suggest that, within immunosuppressed women, the anatomical distribution of HPV-related genital disease could provide clinically meaningful anal risk stratification (4, 13).

The 2024 IANS consensus guidelines endorsed a risk-based approach for ASCC screening, recommending screening for WLHIV, solid organ transplant recipients, and individuals with prior vulvar precancer or cancer, while supporting shared decision-making for selected intermediate-risk populations (11). This approach has recently been adopted by other European and American scientific societies (10, 24). The marked differences in HSIL/AIN prevalence observed across genital disease groups in our cohort support that the anatomical distribution of the HPV-associated genital HSIL, particularly the presence of vulvar and multizone HSIL, should be considered as an additional risk-stratification factor of ASCC among immunosuppressed women. Our findings indicate that the addition of risk factors may confer an increased risk of concurrent anal HSIL/AIN and could help prioritize screening for these patients. Interestingly, women with vulvar HSIL and multizone HSIL—the groups with the highest prevalence of anal HSIL/AIN—tended to be younger than women with isolated cervical HSIL (mean age 43.8 and 43.4 vs. 59.2 years, respectively; p=0.080). Although these findings should be interpreted cautiously given the limited sample size, they raise the hypothesis that the accumulation of HPV-related risk factors may not only increase the likelihood of HSIL/AIN but also identify women who develop clinically relevant disease at younger ages. If confirmed, these findings would support further refinement of current risk-based screening strategies (11) by incorporating the anatomical distribution of genital HPV-related disease in addition to age and underlying immunosuppressive condition. In our previous study on immunocompetent women with genital HPV-associated disease, anal HSIL/AIN was uncommon but clustered in women with multizone HSIL, in whom prevalence exceeded 20% (14). In the present study, conducted in the same clinical setting and using the same diagnostic approach, but focused on immunosuppressed women, the burden of anal HSIL/AIN was substantially greater. This difference was particularly evident in women with isolated vulvar HSIL, in whom HSIL/AIN prevalence increased more than 15-fold, from 3.2% in immunocompetent to 52.6% in immunosuppressed women. Although more modest, we observed a threefold increase in prevalence in women with isolated cervical HSIL (from 6.0% to 18.2%). Together, our findings indicate marked differences in the magnitude of risk increase in immunosuppressed women, depending on the anatomical region involved by the HPV-associated disease. Our findings suggest that the cumulative risk should be considered in future ASCC screening algorithms.

Anal high-risk HPV infection remained one of the strongest independent predictors of HSIL/AIN in both univariable and multivariable analyses. Compared with women with a negative anal HPV test, both anal HPV16 infection (adjusted OR 35.0; 95% CI 3.0–406.4) and infection with high-risk HPV types other than HPV16/18 (adjusted OR 42.8; 95% CI 3.1–591.5) were independently associated with markedly increased odds of HSIL/AIN. Histological HSIL/AIN was detected in 18/35 (51.4%) women with anal HPV16 compared with 13/34 (38.2%) women with HPV types other than 16/18 and 1/23 (4.3%) women with negative anal HPV testing. Together, these findings support the clinical value of anal HPV testing for risk stratification. However, the overlapping confidence intervals and limited sample size preclude conclusions regarding differences in oncogenic risk between individual HPV genotypes.

The concordance between cervical and anal HPV genotype was substantial. Among women with concurrent cervical and anal HPV infection, 85.2% of those with cervical HPV16 also had anal HPV16, 50.0% of the women with cervical HPV18 showed anal HPV18, and 68.4% of the women with cervical HPV other than 16/18 also had anal HPV other than 16/18. These findings are consistent with previous reports describing strong genotype concordance across genital and anal regions and support the hypothesis that HPV autoinoculation across adjacent anogenital sites may contribute to anal HPV acquisition (12, 14, 25).

Screening strategies in our cohort illustrated the expected trade-off between maximizing HSIL/AIN detection and minimizing referrals to HRA. Anal HPV testing alone showed very high sensitivity (97.0%; 95% CI: 84.2-99.9) and negative predictive value (95.7%; 95% CI: 78.1-99.9), but limited specificity (32.8%; 95% CI: 21.8-45.4), resulting in a referral rate to HRA of 77.0%. These figures are broadly consistent with a recent systematic review reporting pooled sensitivity and specificity estimates of 91.1% and 47.1%, respectively, for anal HPV testing in high-risk women (13). In our study, the combined strategy based on positive HPV testing or abnormal cytology achieved 100% sensitivity (95% CI: 89.4-100) and negative predictive value (95% CI: 79.4-99.9) but at the cost of further reducing specificity and increasing referrals, in line with previous reports (26). By contrast, strategies requiring both HPV positivity and abnormal cytology reduced referrals but missed a clinically relevant number of HSIL/AIN cases.

The main limitation of anal HPV testing is its low specificity in a setting of high anal HPV prevalence. This contrasts with our previous study in immunocompetent women from the same setting, in whom HPV testing alone achieved similarly high sensitivity but substantially fewer HRA referrals. Within this cohort, adding anal cytology as a triage test among HPV-positive women appeared to help prioritize referrals for HRA (13, 14, 26). Although its standalone sensitivity was suboptimal, cytology provided clinically meaningful risk stratification among HPV-positive women: HSIL/AIN prevalence increased from 20.0% in those with negative cytology to 80.0% in those with HSIL cytology. These findings support the use of cytology to prioritize HRA referral among HPV-positive women, particularly when HRA capacity is limited, while underscoring that relevant lesions may still occur despite negative cytology.

To our knowledge, this is the first study to evaluate whether the anatomical distribution of HPV-related genital disease improves anal HSIL/AIN risk stratification within an immunosuppressed population. These findings are further strengthened by several methodological features. All participants were evaluated within a single specialized lower anogenital tract unit using the same predefined diagnostic algorithm, minimizing variability in referral, clinical assessment, and management. In addition, the study included systematic assessment of the entire lower anogenital tract, a standardized anal screening protocol incorporating high-risk HPV testing, liquid-based cytology, and high-resolution anoscopy, together with histopathological evaluation using standardized WHO diagnostic criteria. Finally, the inclusion of a broad range of immunosuppressive conditions beyond WLHIV enhances the generalizability of our findings. However, several limitations should also be acknowledged. First, the relatively small number of women with histologically confirmed HSIL/AIN may have limited the multivariable analysis. In addition, although the model was restricted to the main clinically relevant variables associated with HSIL/AIN in the univariable analysis, the number of events was limited in relation to the number of estimated parameters. Therefore, the adjusted associations would require confirmation in larger cohorts. Second, the cross-sectional design precludes assessment of lesion persistence, progression, or risk of subsequent ASCC. Third, this study is subject to potential partial verification bias because women with both negative anal high-risk HPV test and negative anal cytology did not undergo HRA and were classified as negative for HSIL/AIN in screening performance analysis. Although the probability of underlying HSIL/AIN in women with both negative screening tests is expected to be very low, considering the pathogenesis of anal squamous premalignant lesions, almost constantly associated with HPV, and the high sensitivity of HPV test, the absence of systematic histological verification may have led to some overestimation of sensitivity and negative predictive value. Therefore, although the magnitude of this bias is likely to be limited, these diagnostic performance estimates should be interpreted with caution. Finally, the heterogeneity of the immunosuppressed cohort should also be considered when interpreting our findings. Although all women were receiving treatment for their underlying condition, detailed information on specific immunosuppressive or antiretroviral drugs, treatment duration, and CD4 cell counts in WLWH was not systematically recorded. We were therefore unable to assess whether differences on treatment type, duration or severity of immunosuppression influenced the risk of anal HSIL/AIN.

In conclusion, immunosuppressed women with genital HSIL represent a population with a substantial risk of anal HSIL/AIN. The anatomical distribution of genital disease, particularly the presence of vulvar HSIL, appears to provide clinically meaningful risk stratification among immunosuppressed women. In this cohort, anal HPV testing showed high sensitivity and NPV and may represent a useful primary screening approach, with anal cytology potentially helping to prioritize HRA referral among HPV-positive women when HRA capacity is limited. These findings do provide evidence that may contribute to future refinement of risk-based anal cancer screening strategies and to a more efficient allocation of HRA resources.

## Data Availability

The raw data supporting the conclusions of this article will be made available by the authors, without undue reservation.
