# Correction: Stratifying the risk of anal high-grade squamous intraepithelial lesion among immunosuppressed women with genital HPV

**DOI:** 10.3389/fimmu.2026.2023433

**Published:** 2026-10-26

**Authors:** Isabel Matas, Maria Pifarré, Aureli Torné, Adela Saco, Katarzyna Darecka, Esther Guerra, Cristina Martí, Judit Bruch, Ada Cardiel, Natalia Rakislova, Lorena Marimon, Isabet Mayordomo-Bofill, Ingrid Victoria, Silvia Alós, Pere Fusté, Jaume Ordi, Marta del Pino

**Affiliations:** 1 Institute Clinic of Gynecology, Obstetrics, and Neonatology, Hospital Clínic, University of Barcelona, Barcelona, Spain; 2 Institut d’Investigacions Biomèdiques August Pi i Sunyer (IDIBAPS), Barcelona, Spain; 3 Department of Pathology, Hospital Clínic, University of Barcelona, Barcelona, Spain; 4 ISGlobal, Hospital Clínic, University of Barcelona, Barcelona, Spain

**Keywords:** anal intraepithelial neoplasia, high grade squamous intraepithelial lesion (HSIL), human immunodeficiency virus, human papillomavirus, immunosuppressed women

An incorrect **Funding** statement was provided. The correct **Funding** statement reads:

“The author(s) declared that financial support was received for this work and/or its publication. This study was funded by “Instituto de Salud Carlos III (ISCIII)” [Project PI21/00928 to MD; Project PI25/00537 to MD] and co-funded by the European Union, the “Convocatòria Intensificació Interna per als professionals de l’Hospital Clínic de Barcelona” 2024 [to MD], the “Fundació la Marató de TV3 2023” [subproject number 202402–31 to MD] and the MSD Investigator-Initiated Studies Program (MISP). The funders had no role in the design of the study; in the collection, analysis, or interpretation of data; in the writing of the manuscript; or in the decision to publish the results.”

The original version of this article has been updated.

